# P10 Remote monitoring of patients receiving OPAT: a pilot study

**DOI:** 10.1093/jacamr/dlaf239.014

**Published:** 2026-01-05

**Authors:** Rebecca Ritchie, Cheryl Hastie, Michelle Spittal, Hazel Mitchell, Ann L Noble

**Affiliations:** NHS Lanarkshire, UK; NHS Lanarkshire, UK; NHS Lanarkshire, UK; NHS Lanarkshire, UK; NHS Lanarkshire, UK

## Abstract

**Objectives:**

Up to 25% of patients receiving OPAT will develop a complication of their infection or treatment, requiring unplanned assessment or admission. In our institution, patients attending daily for IV antibiotics, or receiving home administration by district nurses, routinely have daily monitoring of their vital signs. However, patients who are self-administering (or having antibiotics administered by a carer) do not have daily observations. In this pilot study, we assessed the advantages and disadvantages of a novel system for remote monitoring of vital signs in the patient’s home.

**Methods:**

A remote monitoring system known as DOCCLA was used. Patients were given equipment for home measurement of blood pressure, pulse, temperature, respiratory rate and oxygen saturation. They were asked to complete observations and a questionnaire daily regarding their symptoms. Results were returned electronically to a team of clinicians who were available 24/7 to respond to abnormal observations or change in symptoms. Where necessary, these clinicians contacted the patient direct and arranged acute interventions.

**Results:**

Fifteen patients on IV antibiotics via OPAT were referred for remote monitoring, of whom 13 submitted observations and questionnaire responses. A median of 17.5% (range 6.6-24.2) of clinical observations were flagged as outside predefined reference ranges. None of these led to urgent in-person clinical review. Patients reported a range of symptoms, including pain, nausea, dizziness, constipation and malaise. No patients required urgent in-person review due to symptoms reported on the daily questionnaire. One patient contacted the clinical team out of hours as she felt unwell. She was referred urgently to a hospital assessment unit but did not require admission. Anonymized patient feedback demonstrated that patients felt reassured by regular monitoring of vital signs and availability of clinicians for advice and support 24/7. Many reported initial apprehension in setting up and using the system, however, were pleased that they were able to do this successfully. Some patients felt anxious at abnormal observation results when they were feeling well. Some logistic issues with the service were also reported.

**Conclusions:**

In this pilot study, patients were generally supportive of the system for daily clinical observations and symptom monitoring and felt reassured by having 24/7 direct access to clinical support, something that most OPAT services are unable to provide. However, flagged observation results were generally non-significant and generated workload for the clinical team and anxiety for patients. Further work is needed to develop a more personalized approach to reference ranges for observations, and to determine which OPAT patient groups are most likely to benefit from remote monitoring.

